# Development of a Low-Barrier, Reimbursable Take-Home Naloxone Program at a Regional Health System

**DOI:** 10.5811/westjem.47387

**Published:** 2025-11-18

**Authors:** Kory S. London, Sejal Patel, Drew Lockstein, Jamal Rashid, Dennis Goodstein, Richard Pacitti, TaReva Warrick-Stone, Frederick Randolph, Alan Cherney, Karen Alexander, Megan Reed

**Affiliations:** * Thomas Jefferson University, Department of Emergency Medicine, Philadelphia, Pennsylvania; † Thomas Jefferson University, Department of Pharmacy, Philadelphia, Pennsylvania; ‡ Thomas Jefferson University, Department of Hospital Medicine, Philadelphia, Pennsylvania; § Thomas Jefferson University, College of Nursing, Philadelphia, Pennsylvania

## Abstract

**Introduction:**

Take-home naloxone (THN) programs in emergency departments (ED) can reduce opioid overdose deaths by providing naloxone directly to at-risk patients before discharge. However, sustainable models that integrate reimbursement and workflow alignment remain limited.

**Methods:**

A reimbursable ED-led THN program was developed across a large regional health system. The program used electronic health record (EHR)–integrated ordering, on-site kit dispensing, and third-party insurance billing when available. Kits were stocked in automated medication dispensing systems and supplemented by city-provided stock for uninsured patients. Pilot outcomes included kits dispensed and reimbursement rates across eight participating EDs.

**Results:**

A total of 2,520 naloxone kits were dispensed across eight EDs between January 2019–December 2024, with a total of 6,551 encounters with decision support prompting naloxone ordering (31.6% of eligible). The proportion of kits reimbursed by insurance rose from 46% in 2019 to 95% by 2025. In total, 89.9% of kits were reimbursed either by insurance or public supply (the rest paid by the hospital system). Kit distribution grew from 99 in 2019 to 702 in 2024, reflecting expanded site participation, improved workflows, and greater staff engagement.

**Conclusion:**

A reimbursable ED-led naloxone program can increase access to life-saving medication for patients at risk of opioid overdose. Integrating take-home naloxone distribution into EHR workflows, leveraging insurance billing, and partnering with public health agencies offers a sustainable, low-barrier model that other health systems can adopt.

## INTRODUCTION

Opioid overdose remains a leading cause of preventable death in the United States, further exacerbated by an increasingly complicated and adulterated illicit drug supply.^1^ Hospital emergency departments (ED) are critical touchpoints for overdose prevention efforts. Being treated at an ED for a non-fatal overdose is a significant predictor of a subsequent fatal overdose, underscoring the need to reach an incredibly vulnerable population of people who use drugs.^2^ Traditionally, patients at risk for opioid overdose might receive a naloxone prescription in the ED to fill at a pharmacy later or may be told how to acquire naloxone on their own in the community. This approach often falls short with one nationwide analysis finding that only about 7% of ED visits for opioid overdose result in a naloxone prescription being issued, and an even smaller fraction are actually filled at an outside pharmacy.^3^ Stigma from requesting this medication in a public setting is likely a contributing factor in this challenge.^4^,^5^

In practice, studies report that only approximately 1 in 5 ED naloxone prescriptions are picked up by patients.^6^ The result is that many high-risk individuals leave the hospital without this critical tool. By contrast, take-home naloxone (THN) programs that dispense naloxone kits directly to patients before discharge can dramatically improve access and uptake. By reducing barriers to getting the medicine into the hands of patients, one program was able to offer naloxone to nearly 50% of eligible overdose patients when kits were given in the ED instead of relying on outpatient prescriptions.^7^ Similarly, a recent multiphase study observed steep improvements in dispensing of naloxone when clinicians hand out kits and even more when all ED staff (nurses, pharmacists, etc) participated.^8^ These data confirm that low-threshold THN models—where naloxone is provided immediately and without out of pocket expense, without extra steps for the patient— can greatly expand access in the ED setting.

Another crucial consideration in hospital naloxone programs is sustainability and cost.^9^ Naloxone medications (especially intranasal formulations) have a significant unit cost, and hospitals must account for staff time to provide overdose education. Traditional insurance reimbursement mechanisms pose challenges: Most insurers will not reimburse a medication dispensed directly by the ED or inpatient unit (since those are billed under facility fees rather than outpatient pharmacy claims). Thus, if an ED simply hands out a kit from floor stock, the hospital may bear the cost unless alternative billing arrangements are in place.

Our health system implemented a THN program leveraging several of the following best practices: integrating distribution into the electronic health record (EHR) for billing when possible; providing kits free of charge at bedside; engaging multidisciplinary staff in the workflow; and using public partnerships for uninsured patients. We describe the development and pilot outcomes of this low-barrier, reimbursable naloxone distribution program in a large regional health system. The primary outcome was the number of naloxone kits dispensed from the EDs. We also discuss how our approach aligns with emerging evidence and share insights to inform similar efforts in other hospitals.

## METHODS

### Study Design and Setting

This study was an implementation science manuscript combined with a retrospective observational pilot study conducted from January 2019–December 2024 in Philadelphia, PA. The study took place across EDs within Jefferson Health, a large regional health system. Initially, four hospitals participated, with four additional hospitals added progressively over the study period, totaling eight dispensing sites by the study’s conclusion.

**Population Health Research Capsule**

What do we already know about this issue?

*Naloxone is a lifesaving medication that can be vital for those who use opioids. Dispensing naloxone after overdose is fundamental harm reduction*.

What was the research question?

*What evidence-based, practical steps can increase access and provision of naloxone from the emergency departments (ED) of a hospital system?*

What was the major finding of the study?

*2,520 naloxone kits were dispensed from eight EDs, from 6,551 eligible encounters (31.6%). The proportion of kits reimbursed by insurance rose from 46% in 2019 to 95% by 2025, with 89.9% overall reimbursed by insurance or public supply*.

How does this improve population health?

*Naloxone from the ED makes communities more resilient to the overdose crisis. By destigmatizing and normalizing harm reduction, health-positive care is possible*.

### Participants

Participants included ED patients identified as at risk for opioid overdose based on predefined criteria, such as presentation for opioid overdose or opioid-related care. Patient selection occurred via automated prompts integrated within the EHR, as well as clinical discretion. We included all eligible patient encounters within participating EDs during the implementation period.

### Intervention

A multidisciplinary team of pharmacists, physicians, and nurses at Jefferson Health designed and implemented a THN program. The core intervention involved placing an order for naloxone kits via the EHR (Epic Systems Corporation, Madison, WI, and Cerner Millenium, Oracle Health, Kansas City, MO), which was automatically routed to the hospital outpatient pharmacy for third-party insurance billing when possible and then dispensed through an automated medication storing/dispensing system (AMDS). Each kit contained two doses of 4 mg of intranasal naloxone as well as graphic and written instructions on use. Additionally, each kit had a sticker-based pharmacy prescription label (see Appendix 1), which was filled out and signed by a clinician or pharmacist to maintain regulatory requirements on medication provision in the ED.

In the hospitals using EPIC EHR, patients with a chief complaint of overdose who had opioid use disorder in their problem list or received an *International Classification of Diseases, 10^th^ Rev*, diagnosis involving F11 codes also received active clinical decision support via a “best practice advisory,” which fires in the disposition navigator, encouraging THN ordering. Naloxone nasal spray kits (two 4 mg Narcan nasal sprays per kit) were pre-stocked in AMDS within the EDs. Clinicians or pharmacists dispensed kits directly from these systems by labeling kits appropriately and handing them to nurses, who then provided the kits to patients at discharge along with brief overdose response education. This included instructions on how to identify an overdose, how to use the naloxone and its mechanism, and how to get additional community-based naloxone.

For insured patients whose claims were accepted, kits were billed as standard outpatient prescriptions; no co-pays were required. In the event a co-pay was required by the insurer, or the patient was uninsured, kits were provided free through a replenishment agreement with the Philadelphia Department of Public Health. The acquisition cost for naloxone kits ranged from free when provided by the city to a maximum of $36.81 per dispensed item if the hospital system paid for the item. Reimbursement was defined as being able to wholly cover the minimum costs of acquiring, storing, and dispensing the medication (approximately $40.00). Members of the outpatient pharmacy staff monitored an EHR naloxone-distribution report for doses dispensed. Staff would submit claims to insurers for reimbursable doses and document the date/time dispensed for city reporting purposes.

### Staff Training and Program Implementation

Staff received ongoing education through in-service training sessions, printed workflow guides, and EHR quick-reference sheets to facilitate consistent program adoption. Hospital-based peer specialists—those with lived experience who help engage and advocate for recovery—and social workers also played an active role in workflow dissemination to front-line caregivers. Implementation data collection included numbers of kits dispensed and insurance reimbursement rates, covering January 2019–December 2024.

### Outcomes and Variables

The primary outcome measured was the number of naloxone kits dispensed at discharge. Secondary outcomes included reimbursement rates, differentiated by insurance coverage or public health-provided supplies. Exposure to the intervention was defined by participation in the ED-led naloxone-dispensing protocol. Selection biases were minimized given the data was acquired and maintained through an automated electronic pharmacy database. Chart abstractors were not blinded to the study hypothesis.

### Statistical Analysis

Quantitative data, including dispensed naloxone kits and reimbursement rates, were analyzed descriptively. We summarized continuous data as totals and percentages, and trends annually and monthly. Analyses were performed using Microsoft Excel (Microsoft Corporation, Redmond, WA), focusing exclusively on descriptive statistics without hypothesis testing or adjustment for confounders. No formal sample-size calculation was conducted due to the pilot nature of this observational implementation study.

### Ethical Considerations/Funding

The institutional review board reviewed the study protocol and deemed it exempt based on its retrospective and de-identified nature. The study reporting follows the STROBE (Strengthening the reporting of observational studies in epidemiology) guidelines for observational studies.^10^ The only external funding for this work was through the Philadelphia Department of Health, which provided dose-for-dose replenishment for any kits that could not be reimbursed through public or private insurers.

## RESULTS

This study was conducted from January 1, 2019–December 31, 2024. Across the entire study period, 2,520 naloxone kits were dispensed systemwide. The EDs that use the Epic EHR (all except the Einstein campuses) recorded a total of 6,551 patients for whom the best practice advisory fired, dispensing 2,073 naloxone kits (31.6%).

**Figure 1:**
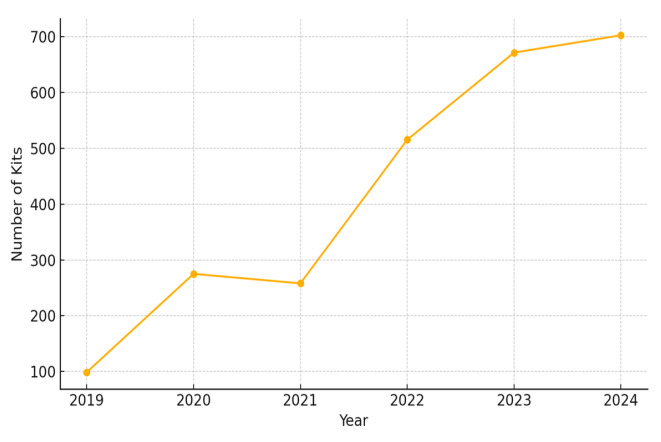
Annual number of take-home naloxone kits dispensed from emergency departments, 2019–2024.

Naloxone distribution increased over time, from 99 kits in 2019 to 702 kits in 2024. This growth coincided with the introduction of EHR-integrated workflows, expansion to new campuses, and enhanced staff engagement. Figure 1 shows the continued rise in monthly dispensing through 2024. The most substantial contributions came from Jefferson Torresdale/Frankford (n=1,166 kits), Thomas Jefferson University Hospital (n=491), and Methodist Hospital (n=418). Newer participating hospitals, Einstein Philadelphia and Einstein Montgomery, dispensed 370 and 77 kits, respectively, despite joining the program mid-course.

Table 1 summarizes naloxone dispensing and reimbursement data by year and site. Across the program, 1,147 kits (43.9%) were reimbursed through third-party insurers (primarily Medicaid), and 1,178 kits (45.1%) were covered through a replacement agreement with the Philadelphia Department of Public Health. The rest were paid by the hospital system as part of operating costs. In total, 2,265 of the 2,520 dispensed kits (89.9%) were reimbursed by either mechanism. No patients were charged directly for naloxone at any point, indicating strong fidelity to the program’s low-barrier and no-cost design. The total cost savings, compared to the hospital system paying for every dispensed dose, was approximately $90,000.

Reimbursement performance improved over time. In 2019, only 46.5% of distributed kits were reimbursed. This increased to 100% by 2021, dipping only briefly during the onboarding of new hospitals to the program, and then exceeded 90% throughout 2023 and 2024.

## DISCUSSION

### Interpretation/Summary of Findings

The implementation of an ED-led take-home naloxone program was associated with increases in naloxone distribution, improved reimbursement over time, and broad uptake across a diverse group of hospitals. The intervention was built iteratively across the system and achieved sustainable fidelity through a focus on evidence-based insight and incremental improvement. This pilot demonstrates the feasibility and scalability of a hospital-based, reimbursable THN program. By integrating naloxone distribution into the existing ED workflow and EHR system, we were able to use third-party insurance billing when available and minimize additional steps for staff. The provision of free kits via the Philadelphia Department of Health ensured equitable access when insurance did not cover the cost. We observed year-over-year growth in naloxone dispensing across our EDs, which suggests increasing staff buy-in and patient acceptance over time.

### Comparison to Previous Studies

Notably, our experience aligns with broader evidence that bringing naloxone directly to the bedside improves access and can lead to overdose reversals in the community. Prior studies confirm that patients are far more likely to receive and use naloxone when it is dispensed on-site in the ED, rather than simply prescribed.^7^,^11^,^12^ Many patients, however, still did not receive this intervention. This gap underscores the need for equity-focused ED protocols to ensure vulnerable groups are not overlooked in naloxone provision.^13^ National data shows that THN is under-prescribed in EDs across the country, with even lower rates among women, older or younger patients, non-English speakers, and certain racial/ethnic groups.^14^,^15^ Such disparities highlight areas for opportunity for improvement in equitable naloxone distribution from the ED.

### Strength and Clinical Interpretation

Our THN initiative adds to the growing evidence that health system-based programs can successfully reduce barriers and reach vulnerable patients who might otherwise not obtain naloxone. We demonstrated a model in which clinical workflows and billing mechanisms were leveraged to sustain the program, supplemented by public health partnerships.

### Clinical and Research Implication

Geographic barriers contributing to naloxone access inequity also exist; a spatial study noted that underserved areas are often at highest risk of having an overdose occur.^16^ This is of concern as those experiencing homelessness are at greater risk for both fatal and non-fatal overdoses.^17^,^18^ An ED-based program, by directly handing out kits, could help circumvent these “naloxone deserts” and reach patients who might otherwise have no access. Multiple studies reiterate that ED access is a strong first step in providing this community service.^19^–^20^ Further addressing these issues in our THN naloxone program, for example, through stigma-reduction training and involving people with lived experience in program design, was essential to help ensure we reached as many individuals as possible. Future studies should continue to assess patient and clinician barriers to THN dispensing to vulnerable patients.

Additional clinical opportunities include providing THN to at-risk patients upon discharge from inpatient units, to individuals who are prescribed opioids but do not have an opioid use disorder, and exploring providing additional harm reduction supplies (eg, fentanyl test strips) at discharge. By making THN a routine part of emergency care, hospitals can play a crucial role in preventing overdose deaths and connecting high-risk patients to further services, ultimately contributing to a broader culture of harm reduction in healthcare.

## LIMITATIONS

This study was conducted as a pilot-program evaluation at a single regional health system, which limits generalizability. Our data reviewers were not blinded to our hypotheses. Our outcome data are primarily process-oriented (number of kits dispensed and reimbursement rates); we were not able to directly track demographics, the use of distributed naloxone kits, or the downstream impact on overdose events and patient behavior after ED discharge. Follow-up with patients was not performed. Additionally, while we infer that greater naloxone dispensing likely prevented some overdoses, we cannot definitively attribute any changes in overdose outcomes to our program alone. Another limitation is that our program’s success was facilitated by unique resources such as an internal outpatient pharmacy and a city-supplied naloxone stock, which may not be available in all hospital settings.

Finally, cultural change among staff, while anecdotally observed, was not formally measured. Resistance or stigma in other settings could pose barriers that were addressed in our system via strong leadership support. Despite these limitations, we believe our findings demonstrate practical strategies that can be adapted elsewhere, and they highlight the need for further study (ideally multicenter and with patient follow-up) to fully quantify the benefits of ED THN distribution programs.

## CONCLUSION

Hospital-based and ED-based take-home naloxone distribution programs have emerged as a crucial component of the overdose response, offering a “low-barrier” safety net to individuals at high risk for opioid overdose. Our experience and the recent literature show that these programs are feasible to implement and can greatly increase the reach of naloxone into the community. Key elements for success include making naloxone readily accessible at patient discharge (preferably at no out-of-pocket cost), embedding the process into standard clinical workflows (with EHR support and multidisciplinary staff involvement), securing sustainable funding or reimbursement, and educating staff to foster a supportive, harm reduction-oriented culture. Continued innovation will be important to determine which workflow refinements or educational approaches yield the highest uptake, and how these programs can best link patients to long-term recovery resources. Future work should also evaluate longer term patient outcomes from such programs, including overdose mortality rates, subsequent treatment engagement, and cost effectiveness.

## Supplementary Information



## Figures and Tables

**Table 1:** Naloxone kit distribution totals and reimbursement outcomes by hospital site from 2019–2024.

Year/Month	TJU-CC	MHD	JNE	JHN	EinsteinP	EinsteinM	Abington	Lansdale
2019 Summary	35	36	28	0	--	--	--	--
2020 Summary	80	52	142	1	--	--	--	--
2021 Summary	71	68	119	0	--	--	--	--
2022 Summary	121	82	189	1	58	14	36	14
2023 Summary	88	83	309	0	140	30	16	5
2024 Summary	92	90	352	0	120	28	14	6
Total	491	418	1,166	2	370	77	67	21

Year/Month	Total # Dispensed	3rd Party Reimbursement	Replacement Dose Provided by City	Total Reimbursed (%)
2019 Summary	99	33	13	46 (49)
2020 Summary	275	100	145	245 (89)
2021 Summary	258	149	109	258 (100)
2022 Summary	515	169	229	398 (77)
2023 Summary	671	314	306	620 (92)
2024 Summary	702	283	385	668 (95)
Total	2,520	1,078	1,187	2,265 (90)

*TJU-CC*, Thomas Jefferson University Hospital; *MHD*, Jefferson Methodist Hospital; *JNE*, Jefferson Torresdale and Frankford Hospitals; *EinsteinP*, Jefferson Einstein Philadelphia Hospital; *EinsteinM*, Jefferson Einstein Montgomery Hospital; *Abington*, Jefferson Abington Hospital; *Lansdale*, Jefferson Lansdale Hospital
