# Supplementary material for: Development of a Low-Barrier, Reimbursable Take-Home Naloxone Program at a Regional Health System
Source: West J Emerg Med. 2025 Nov 18;26(6):1605–10. doi: 10.5811/westjem.47387 (PMC12698159; doi:10.5811/westjem.47387)
Supplement: Supplementary file 1 [file wjem-26-1605-s001.docx]

**Appendix 1: Example Naloxone Prescription Label**

Date________________

Patient Name_______________________________________

**Narcan 4mg Nasal Spray (naloxone)**

Use as Directed

#1 Kit (2 Pack) No refills

Provider Name__________________________________

Provider Signature_______________________________

Jefferson Apothecary

111 S 11^th^ St

Philadelphia, PA 19107

Phone: 215-955-8845

FILL OUT LABEL PRIOR TO DISPENSING TO PATIENT
